# An Exploration of Learning Results and Curricula Gaps Within an Online Grandfamilies Certificate Program

**DOI:** 10.1093/geroni/igaa057.231

**Published:** 2020-12-16

**Authors:** Jennifer Crittenden

**Affiliations:** University of Maine, Bangor, Maine, United States

## Abstract

Professionals who work with grandparents raising grandchildren have cross-cutting training needs that span content in gerontology, social services, child welfare and program development. To address these needs, a unique, asynchronous, online continuing education program was launched by the UMaine Center on Aging. To-date the program has 177 individual program completers with learners from across the U.S. and Hong Kong that are affiliated with a diverse set of organizations and perform a wide range of professional and lay functions. Participant data indicate that the program has appealed to a wide variety of learners including participants who serve caregivers generally (60.8%) and grandparents raising grandchildren specifically (81%). A small majority (55.6%) of the agency-based learners reported serving, on average, more than 40 grandfamilies annually. Self-reported learning levels were notable ranging from a mean low of 3.46 out of 4 points (N = 157, SD = 0.59) for the volunteer recruitment and mentorship programming module to a mean high of 3.79 (N = 167 ,SD = 0.45) for the caregiver self-care module. Evaluation results from the first seven learner cohorts underscore the efficacy of program content as well as the utility of performing an initial program needs assessment to guide curriculum development. Practice implications for future continuing education efforts targeting grandfamilies professionals and lay leaders include: the need for easily accessible online education in combination with supplemental training opportunities addressing topics such as the long-term impact of substance use disorder and trauma combined with locally relevant content on grandfamilies and legal resources.

